# Psychosis, Socioeconomic Disadvantage, and Health Service Use in South Australia: Findings from the Second Australian National Survey of Psychosis

**DOI:** 10.3389/fpubh.2015.00259

**Published:** 2015-11-20

**Authors:** Shaun Sweeney, Tracy Air, Lana Zannettino, Cherrie Galletly

**Affiliations:** ^1^Discipline of Psychiatry, University of Adelaide, Adelaide, SA, Australia; ^2^School of Nursing and Midwifery, Flinders University, Adelaide, SA, Australia

**Keywords:** psychosis, socioeconomic disadvantage, health, poverty, health service delivery

## Abstract

The association between mental illness and poor physical health and socioeconomic outcomes has been well established. In the twenty-first century, the challenge of how mental illnesses, such as psychosis, are managed in the provision of public health services remains complex. Developing effective clinical mental health support and interventions for individuals requires a coordinated and robust mental health system supported by social as well as health policy that places a priority on addressing socioeconomic disadvantage in mental health cohorts. This paper, thus, examines the complex relationship between socioeconomic disadvantage, family/social supports, physical health, and health service utilization in a community sample of 402 participants diagnosed with psychosis. The paper utilizes quantitative data collected from the 2010 Survey of High Impact Psychosis research project conducted in a socioeconomically disadvantaged region of Adelaide, SA, Australia. Participants (42% female) provided information about socioeconomic status, education, employment, physical health, contact with family and friends, and health service utilization. The paper highlights that socioeconomic disadvantage is related to increased self-reported use of emergency departments, decreased use of general practitioners for mental health reasons, higher body mass index, less family contact, and less social support. In particular, the paper explores the multifaceted relationship between socioeconomic disadvantage and poor health confronting individuals with psychosis, highlighting the complex link between socioeconomic disadvantage and poor health. It emphasizes that mental health service usage for those with higher levels of socioeconomic disadvantage differs from those experiencing lower levels of socioeconomic disadvantage. The paper also stresses that the development of health policy and practice that seeks to redress the socioeconomic and health inequalities created by this disadvantage be an important focus for mental health services. Such health policy would provide accessible treatment programs and linked pathways to illness recovery and diminish the pressure on the delivery of health services. Consequently, the development of policy and practice that seeks to redress the socioeconomic and health inequalities created by disadvantage should be an important focus for the improvement of mental health services.

## Introduction

Socioeconomic disadvantage is associated with a higher prevalence of and a higher mortality from most diseases, particularly the major chronic diseases such as heart disease (1, 2). The concept of socioeconomic disadvantage can apply to individuals or populations who reside in low-income circumstances, and who struggle to supply themselves and their families with food, clothing, and shelter. This disadvantage can take multiple forms, including limited job security, poor social networks, low self-esteem, poverty, and fatalism (3). As socioeconomic disadvantage can also include difficulties in accessing government income and social supports, such disadvantage is heightened for people with psychosis whose complex health needs require access to such services. Furthermore, the links between psychosis and socioeconomic disadvantage have been identified across diverse cultural, social, and demographic contexts. Research has now established a clear relationship between poverty and psychosis (4), prevalence rates of schizophrenia (5), and rates of admission for schizophrenia (6). Additionally, people with a mental illness can experience lower levels of employment than the general population (7). With regard to clinical characteristics that predict utilization of services, the most common finding has been that psychosis is linked to a higher rate of utilization of specialist services (8, 9). Given this, there is a need for information to enable the planning of and resource allocation for services where people with a psychotic illness present (10).

There is extensive research examining the links between psychosis and poor health and social outcomes. However, there has been less research focusing on the relationship between psychosis, socioeconomic disadvantage (including homelessness, poverty, and social isolation), and the use of health services (11). For example, while psychosis can limit income capacity, it is less clear how this affects the level and type of health service usage rates of social engagement and the ability to effectively manage illness symptomatology. Consequently, attempts to determine the most appropriate social policy, service practice mandates, and praxis in this area have lacked coherence. The relationship between the influence of socioeconomic disadvantage and the increase in the utilization of community services, such as mental health facilities, needs to be examined further (12) and the causal links between psychosis and poor socioeconomic outcomes require further examination to strengthen the knowledge base regarding these links. Previous research has attempted to improve services that address the challenge of poor health and socioeconomic participation among persons with mental illness but have frequently failed to recognize that the experience of people with mental illness is often contextualized in disadvantaged social settings (13). Additionally, the use of health and social services among psychosis populations can differ according to their socioeconomic status (14). Therefore, understanding the relationship between the lived experience of psychosis and socioeconomic disadvantage has the potential to influence the development and implementation of health and social policy initiatives.

The contribution of this paper to the field of mental health is twofold. First, utilizing quantitative data from the Survey of High Impact Psychosis (SHIP) project, the paper presents an overview of health status and level of socioeconomic disadvantage in a regionally representative population with a diagnosis of a psychotic illness. Second, the paper examines the relationship between socioeconomic disadvantage and family/social supports, physical health, and, consequently, health service utilization.

## Materials and Methods

### Subjects

The data for this study were collected during the SHIP research project in the northern suburbs of Adelaide, SA, Australia (15). The northern suburbs^1^ of Adelaide, SA, Australia, have 226,654 residents in a geographical area of 814 km^2^. The population age structure almost equals the national figures. Rates of single-parent families are higher than the national average; unemployment is higher while labor force participation is slightly lower. Qualifications beyond high school are lower in this catchment area (45.6%) compared with the national average (56.2%). Trained interviewers, who were mental health clinicians and had worked in the local mental health services, conducted all of the interviews.

### Assessments

Participants for the study were randomly selected from people who have been in contact with a mental health service or a non-government organization funded to provide mental health services. The SHIP projected utilized a psychosis screener to identify potential participants. Potential participants were screened for psychosis between 1st and 30th of April 2009 (16). The psychosis screener identified potential SHIP participants who were positive for psychosis on the basis of their contact(s) with mental health services or who recorded ICD-10 diagnosis of psychosis. The psychosis screener identified 1825 adults aged 18–64 years who were residents in the South Australian postcode catchment area. Potential participants needed to have been in contact with public mental health services in the 12 months prior to the survey to be eligible to participate in the SHIP study. The exclusion criteria included severe cognitive impairment, the inability to comprehend English sufficiently to complete the interview without the use of an interpreter. Attempts were made to recruit all of these potential participants. Eight hundred and three were unable to be contacted due to change of personal circumstance (e.g., moved away from the catchment area, changed their phone number, were non-responders to phone/mail contact or did not attend the SHIP interview), 16 were known to have died, 33 did not meet inclusion criteria due to inability to sufficiently communicate in English, 42 did not have capacity to give informed consent, and 507 refused. Potential participants were identified during the SHIP census period and in total 402 participants were interviewed.

The SHIP interview schedule consisted of 32 modules and asked participants about psychopathology, substance use, physical health, functioning, disability and quality of life, education, employment, accommodation, and childhood adversity. The social, health, and economic profile information of participants consisted of, but were not limited to, demographic status, socioeconomic and psychosocial status, health and physical functioning, diagnosis, and symptomatology. Diagnoses were made using the Diagnostic Interview for Psychosis (DIP) (17). The DIP contains selected interview questions and probes from the WHO Schedules for Clinical Assessment in Neuropsychiatry (18) mapped onto the 90 diagnostic items of the operational criteria checklist for psychotic and affective illness (19). The DIP measured both lifetime and current illness symptoms for psychosis. A computer algorithm provided the diagnostic classification in accordance with ICD-10, DSM-IV, and other criteria on the basis of the DIP scores. This reduced the subjective bias in the interpretation of symptoms and signs (20). Comparison of screening data for interviewed participants and those selected for interview but not participating for any reason indicated no systematic selection biases. Both groups were alike in terms of sex (60% of those interviewed were male compared to 62% of those selected but not interviewed) and age group (44% of those interviewed were aged 18–34 years at the time of screening compared with 43% of those not interviewed). The psychosis screening profiles for both groups were similar, indicating no marked differences in terms of lifetime symptom profiles based on the screener items. Further information regarding the method of the SHIP research project is detailed in Morgan et al. (15).

The Australian Bureau of Statistics (ABS) Socio-Economic Indexes for Areas (SEIFA) and the Index of Relative Socio-Economic Advantage and Disadvantage (IRSAD) have been utilized in this paper to highlight the social and economic status of the research participants. The SEIFA data are used by the ABS to categorize areas in Australia according to relative socioeconomic advantage and disadvantage. These SEIFA indexes are based on information from the five-yearly ABS census. The SHIP project obtained every participant’s suburb and the percentiles and utilized the SEIFA data as a proxy measure of socioeconomic disadvantage. Additionally, the IRSAD was used to summarize information about the economic and social conditions of the SHIP cohort within its catchment area, including both relative advantage and disadvantage measures. A low score on the SEIFA IRSAD can be indicative of relatively greater disadvantage and a lack of advantage in general.

### Statistical Analyses

Statistical analyses were performed using Stata, version 12 (StataCorp. 2011. Stata Statistical Software: Release 12. College Station, TX, USA: StataCorp LP). A two-step model building procedure was used to determine variables associated with the SEIFA IRSAD. In the first step, we used univariate analyses (linear regressions) to examine whether the percentile measure of the IRSAD was associated with socio-demographic, physical health, social, and health service utilization measures. In the second step, we used multivariate regressions, including only variables that were associated with the SEIFA IRSAD at α ≤ 0.10 in the first step.

### Ethics Statement

The appropriate institutional ethics committees approved the study and all participants gave written informed consent. Ethics approval for this research was obtained from the Human Research Ethics Committee of the Queen Elizabeth Hospital (protocol number: 2009179).

## Results

Table 1 illustrates demographic and lifestyle factors from the SHIP cohort. The SHIP sample recruited in South Australia comprised 402 participants who screened positive for psychosis. There were 168 female participants (42%) and the mean age of the sample was 38.5 (SD = 10.6) years. One-fifth of the sample (20.9%) was currently married or in a *de facto* relationship. The average age that the sample left school was 16 (SD = 1.3) years, while 43% reported having a post-school qualification. The main source of income for 92.5% of the sample was a government pension. In the year prior to interview, 23.4% were in paid employment; however, this figure was reduced to 16.2% for paid employment in the past week. Seventy-two percentage (*n* = 290) of the sample reported being a current smoker. One hundred and sixty-nine participants (42%) reported a lifetime history of alcohol abuse or dependence, while 47.8% had a lifetime history of illicit drug abuse or dependence. Twenty-three percentage of the sample had been victims of violence in the previous year, while 10% had been charged for committing an offense. Table 1 also highlights the breakdown of the DIP diagnoses. The majority of the sample was diagnosed with schizophrenia (31.9%) and schizoaffective disorder (30.7%). No significant relationships were found between any of these demographic and lifestyle factors and the SEIFA IRSAD.

**Table 1:** **Socio-demographic and lifestyle data from the SHIP sample**.

	Ship sample (*n* = 402)	β coefficients (95% CI)	*p*-Value
**Age and gender**
Female (*n*, %)	168 (41.8%)	0.40 (-4.92, 5.72)	0.88
Age (mean ± SD, years)	38.5 ± 10.6	-0.13 (-0.38, 0.11)	0.29
**Education**
Age left school (mean ± SD, years)	16.0 ± 1.3	0.86 (-1.18, 2.90)	0.41
Post school qualification (*n*, %)	174 (43.3%)	5.12 (-0.15, 10.39)	0.06
**Marital status (*n*, %)**
Married/*de facto*	84 (20.9%)	-0.13 (-6.58, 6.33)	0.97
**Income and employment (*n*, %)**			
Main income source: pension	370 (92.5%)	-0.91 (-19.0, 0.86)	0.07
Paid employment (past year)	94 (23.4%)	4.71 (-1.47, 10.90)	0.14
Paid employment (past week)	65 (16.2%)	2.47 (-4.7, 9.6)	0.50
**Smoking, drugs, and alcohol (*n*, %)**
Lifetime alcohol abuse/dependence	169 (42.0%)	-2.72 (-8.03, 2.59)	0.31
Lifetime illicit drug abuse/dependence	192 (47.8%)	-0.31 (-5.57, 4.94)	0.91
Current smoker	290 (72.7%)	1.01 (-4.89, 6.91)	0.74
**Victimization and offending (*n*, %)**			
Victim of violence	93 (23.1%)	-2.71 (-8.93, 3.51)	0.39
Charged with an offense	40 (10.0%)	2.28 (-6.49, 11.04)	0.61
**ICD 10 diagnosis**
Schizophrenia	128 (31.9%)	1.00 (-4.6, 6.6)	0.73
Schizoaffective	123 (30.7%)	2.85 (-2.8, 8.5)	0.33
Bipolar, mania	70 (17.5%)	2.04 (-4.9, 9.0)	0.56
Depressive psychosis	18 (4.5%)	-12.1 (-24.8, 0.49)	0.06
Delusional and other non-organic psychoses	25 (6.2%)	-6.85 (-17.7, 4.0)	0.22
Severe depression	37 (9.2%)	-1.62 (-10.7, 7.5)	0.73

### Physical Health

The mean BMI for the sample was 30.3 (SD = 7.7), while the average waist circumference of the sample 105.5 cm (SD = 19.3) (Table 2). Over half of the sample (51.7%) met criteria for metabolic syndrome according to International Diabetes Federation criteria (21). Twenty-four percentage already had cardiovascular disease or were at high risk for a cardiovascular event in the next 5 years based on the Framingham risk equation (22). The sample also had elevated levels of diastolic and systolic hypertension (50.5 and 40.1% respectively), cholesterol (51.2%), triglycerides (49.1%), and glucose (23.0%) that put them at risk of subsequent cardiovascular disease. A significant inverse relationship was found between the SEIFA IRSAD and both BMI and waist circumference, with lower socioeconomic advantage being associated with higher BMI and waist circumference.

**Table 2:** **Physical health of the SHIP sample**.

	Ship sample (*n* = 402)	β coefficients (95% CI)	*p*-Value
BMI (mean ± SD)	30.3 ± 7.7	−0.34 (−0.7,−0.01)	0.049
Waist circumference (mean ± SD, cm)	105.5 ± 19.3	−0.16 (−0.30,−0.02)	0.023
Metabolic syndrome (*n*, %)	165 (51.7%)	0.48 (−5.5,6.4)	0.87
CVD risk (*n*, %)
Low	223 (66.2%)	0	–
Medium	19 (5.6%)	−0.66 (−13.4,12.1)	0.92
High	13 (3.9%)	−9.76 (−24.9,5.4)	0.21
Already has CVD	82 (24.3%)	−1.59 (−8.5,5.3)	0.65
Diastolic hypertension (≥85 mmHg)	203 (50.5%)	0.43 (−4.8,5.7)	0.87
Systolic hypertension (≥130 mmHg)	161 (40.1%)	−2.01 (−7.4,2.2)	0.46
Elevated cholesterol (*n*, %)	169 (51.2%)	2.16 (−3.7,8.1)	0.47
Elevated triglycerides (*n*, %)	162 (49.1%)	−3.75 (−9.6,2.1)	0.21
Elevated glucose (*n*, %)	76 (23.0%)	0.96 (−6.0,8.0)	0.79

### Social Networks and Support

A large percentage of participants (71.6%) reported frequent contact with their family (Table 3). While most participants (87.1%) reported having someone to rely on and over two-thirds reported that they had at least one person they could confide in, nearly half of the sample (49.3%) felt they needed or wanted more friends and reported experiencing loneliness (81.6%).

**Table 3:** **Social contact in the SHIP sample**.

	Ship sample (*n* = 402)	β coefficients (95% CI)	*p*-Value
Daily contact with family	287 (71.6%)	6.54 (0.8,12.3)	0.027
Someone to rely on	350 (87.1%)	6.80 (−0.99,14.6)	0.087
No-one to confide in	72 (18.1%)	−5.51 (−12.3,1.30)	0.11
Wants more friends	198 (49.3%)	0.78 (−4.5,6.0)	0.77
Has no friends	48 (12%)	−4.96 (−13.1,3.1)	0.23
Experienced loneliness	315 (81.6%)	−4.82 (−11.8,2.1)	0.17
Dysfunction in socializing	190 (47.3%)	−6.05 (−11.3,−0.8)	0.023
**In the last 12 months…**
Experienced stigma	145 (36.2%)	0.29 (−5.2,5.8)	0.92
Fear of experiencing stigma (*n* = 145)	81 (56.6 %)	0.85 (−8.2,9.9)	0.85
Experience of stigma prevented social participation (*n* = 145)	77 (53.1 %)	7.24 (−1.8,16.2)	0.11

Many participants appeared to experience difficulties in maintaining positive social and emotional relationships. For example, 47.3% reported dysfunction in socializing and 12.0% reported having no friends. A significant relationship was found between the SEIFA IRSAD and daily contact with family, with higher socioeconomic advantage being associated with greater contact with family. A significant inverse relationship was found between the IRSAD and dysfunction in socializing, with lower socioeconomic advantage being associated with greater social dysfunction.

The experience of stigma and/or discrimination in this sample was substantial. One hundred forty-five (36.2%) believe that they had experienced stigma or discrimination because of mental illness in the 12 months prior to interview. Of those 145, over half (*n* = 81, 56.6%) reported that fear of stigma or discrimination had stopped them from doing the things they had wanted to do. Furthermore, the experience of stigma and/or discrimination in the 12 months prior to interview prevented 77 (53.1%) of participants from engaging in social activities.

### Health Service Use

Health service utilization in the year prior to interview by participants was higher than for the general population (23), and they primarily relied on the public system for mental health services (Table 4). Forty-one percentage of the sample reported having a mental health-related inpatient admission in the previous year, while 42% reported attending an emergency department specifically for a mental health reason. Twenty-two percentage reported at least one involuntary inpatient admission. However, despite 89.3% using outpatient or community clinic services, only 43% reported having a case manager in the public health services. General practitioners (GPs) also bore the majority of health care provision with nearly 63% of participants reporting that they had visited a GP in the previous year for a mental health-related reason. Emergency department attendance for mental health reasons was found to be significantly associated with lower levels of socioeconomic advantage.

**Table 4:** **Health service utilization in the SHIP sample**.

	Ship sample (*n* = 402)	β coefficients (95% CI)	*p*-Value
Inpatient admission, any	209 (52.0%)	−2.00 (−7.2,3.3)	0.46
Mental health	165 (41.0%)	−2.66 (−8.0,2.7)	0.33
Physical health	72 (17.9%)	2.51 (−5.0,10.0)	0.51
Number of inpatient admissions in past year (mean ± SD)	1.01 ± 1.9	−1.16 (−2.5,0.20)	0.095
Involuntary admission	90 (22.4%)	−0.49 (−6.8,5.8)	0.88
Community treatment order	66 (16.4%)	1.60 (−5.5,8.7)	0.66
Emergency department attendance	230 (57.2%)	−3.39 (−8.7,1.9)	0.21
Mental health	169 (42.0%)	−5.36 (−10.6,−0.06)	0.047
Physical health	100 (24.9%)	−0.02 (−6.1,6.0)	0.99
Outpatient/community clinic contact	359 (89.3%)	−4.10 (−12.6,4.4)	0.34
Home visit, any	208 (51.7%)	0.27 (−5.0,5.5)	0.92
Crisis related	47 (11.7%)	−1.66 (−9.8,6.5)	0.69
Routine visit	165 (41.0%)	3.04 (−2.3,8.4)	0.26
Case manager (public health services)	173 (43.0%)	2.07 (−3.2,7.4)	0.44
GP visits, any	368 (91.5%)	1.83 (−7.6,11.3)	0.70
Mental health	252 (62.7%)	4.51 (0.9,9.9)	0.10
Physical health	298 (74.1%)	−1.09 (−7.1,4.9)	0.72
Number of GP visits (mean ± SD)	12.4 (16.2%)	−0.21 (−2.7,2.3)	0.87

### Relationship Between Social Advantage and Heath and Social Outcomes in a Psychosis Population

Table 5 displays the results of the final multivariate linear regression model. Only the significant variables were retained. BMI, daily family contact, emergency department attendance for mental health reasons, and GP attendance for mental health reasons were significantly associated with the SEIFA IRSAD. Higher BMI and self-reported emergency department attendance for mental health reasons were associated with lower socioeconomic advantage, while greater daily family contact and self-reported GP attendance for mental health reasons were associated with higher levels of socioeconomic advantage.

**Table 5:** **Predictors of socioeconomic disadvantage in people diagnosed with a psychotic illness**.

	β coefficients (95% CI)	*p*-Value
BMI (cm)	−0.34 (−0.68,−0.01)	0.048
Daily contact with family	6.95 (1.2,12.7)	0.019
ED attendance for mental health	−6.25 (−11.6,−0.9)	0.022
GP visit for mental health	6.6 (1.1,12.1)	0.019

## Discussion

This paper’s analysis of the SHIP data illustrates the complex relationship between psychosis, socioeconomic disadvantage, and poorer physical health outcomes. Broader socio-demographic and lifestyle factors, such as employment, income source, educational qualifications, and alcohol and substance abuse/dependence were not significantly associated with SEIFA IRSAD. Mental health service usage for those with higher levels of socioeconomic disadvantage differs from those experiencing lower levels of socioeconomic disadvantage. For example, self-reports of emergency department use in the context of mental health crisis was evident in the cohort with higher levels of socioeconomic disadvantage, as was their lack of engagement with a GP for their management of mental health. Overall, the cohort reported high rates of physical health comorbidity as well as high levels of substance use (see Table 4). Furthermore, socioeconomic disadvantage appears to have exacerbated the poor physical health of participants in the SHIP cohort. Over 51.7% of the study cohort met criteria for metabolic syndrome participants with a body mass index in the overweight or obese range. In comparison, data from the 2007 National Survey of Mental Health and Wellbeing indicate that 34.2% of the general population are overweight and 21% obese (24). Furthermore, as highlighted in Table 1, in the year prior to interview, 23.4% of participants were in paid employment while this figure was reduced to 16.2% for paid employment in the past week. This indicates the transient and short-term nature of employment for this sample. In people with psychosis, poor physical health can compound the burden associated with mental illness and diminish the capacity to establish meaningful vocational and social roles in their communities. Over two-thirds of the participant group smoked cigarettes daily (72.7%). This exceeds the rates of smoking in the general population: 20.4% for males, 16.4% for females (25). While smoking usage rates are consistent with other studies in populations diagnosed with psychosis (26), these results point to a range of health and economic factors that represent challenges for individuals and health services alike.

Unemployment in psychosis populations is common, with a corresponding increase in social stigma (7). As Table 3 demonstrates, participants in the research experienced ongoing stigma related to their mental illness. The ongoing challenge of managing a psychotic illness, particularly schizophrenia, can preclude people from managing the demands of employment and general economic participation. Consequently, poverty can exclude individuals from access to the social opportunities and economic participation enjoyed by others. In addition to these socioeconomic and psychosocial consequences, stigma can also decrease the likelihood that people with a mental illness looking for mental health care (27). Thus, perceptions of stigma about mental illness can have an adverse effect on the lives of people with a mental illness (27, 28). Thus, the combined effects of poverty and social disadvantage are reflected in social exclusion, which can be a reality in the lives of people with a psychotic illness. These effects can potentially hinder the recovery from psychosis and preclude individuals from establishing meaningful social roles.

Psychotic illnesses, such as schizophrenia, are recognized as particularly disabling disorders, which can have poor outcomes for individuals and their families (29). Those with psychosis can often experience a reduction in their quality of life and impaired social functioning (30). Psychosis is associated with multiple social disabilities in work, study, independent living, interpersonal relations, and self-care, and serious disability in functioning is one of the core features of the DSM-V diagnosis of schizophrenia (31). Unemployment in psychosis populations is also common, with a corresponding increase in social stigma (7). Thus, psychosis is associated with increased risks of poverty. The relationship between socioeconomic factors and health outcomes are likely to be significant in determining the use of mental health and social services. The relationship between poverty, social disadvantage, and poor health outcomes has been firmly established in an extensive body of research (32). The health and socioeconomic challenge of psychosis appears to have prevented a significant number of SHIP participants from engaging in healthy physical and social functioning, and economic participation in daily life. This includes engaging in paid employment or participating in other social and economic roles. Hence, psychosis can be viewed as an important factor in determining social and economic disadvantage for this group.

For primary care settings, working with populations, experiencing psychotic illness and the complex health and social difficulties the illness presents, has some challenges. The greater use of emergency department usage, primarily for illness management and/or crisis intervention by those with higher levels of socioeconomic disadvantage, highlights the complexity of this issue. In Australia, hospital emergency departments are not mandated to provide intensive, long-term clinical input other than addressing immediate psychosis symptomatology. For those living in poverty with limited access to family or social supports, emergency departments are the primary source of medical support instead of GPs or other preventative mental health services. Additionally, the greater risk of comorbid illness as a result of poor physical health (e.g., higher BMI or cardiovascular disease) can exacerbate the pressure on services to provide effective health outcomes, particularly when access to preventative health care (e.g., GPs) is restricted by socioeconomic disadvantage. Furthermore, psychosis can predispose a person to increased risks of social disadvantage and poverty through a lack of access to employment, low educational attainment, poor psychological health, social isolation and the risk of social stigma, and/or poor physical health. A range of psychosocial influences can also affect how the illness is manifest. Furthermore, well-documented negative social and cultural perceptions about mental illness create stigma and intensify social and community discrimination. This may affect how people with psychosis can socioeconomically engage in their community.

Evidence from the mental health field indicates that people with psychosis predominantly live in poverty and experience social disadvantage (33, 34). The relationship between poverty, social disadvantage, and poor health outcomes has been firmly established in an extensive body of research (32). For example, a large United Kingdom survey of 8191 adults conducted by Weich and Lewis (35) established a positive relationship between income inequality and mental illness. Research has also confirmed that social disadvantage and poverty are more strongly related to schizophrenia and other psychotic disorders than other mental illnesses (36). Moreover, psychotic illnesses such as schizophrenia often become manifest at a time of critical importance in social development, educational attainment, and employment seeking (37). Low levels of employment (7), diminished social mobility (35), low capacity for productivity (38), limited educational attainment, and poor physical health (39) can create social and economic isolation. This is not only isolated to the singular experience of the individual, but can also occur within a familial environment. For example, in a Swedish study of migrant families, Hjern et al. (40) examined factors related to social adversity, such as parental unemployment, single-parent household, urban residence, adults receiving social welfare benefits, housing, and parental social status of people with psychosis. The study compiled rates of psychoses for adult and youth first-generation migrant cohorts. When rates for these groups were compared to native Swedes and adjusted for household indicators of social adversity, a sizable proportion of the elevated rates of schizophrenia in the adult group, and to a lesser extent in the youth group, was attributed to social and economic disadvantage (41).

The relationship between social disadvantage and poor health status is clear. People who have a psychotic illness exhibit higher rates of obesity (42), poor physical health comorbidity (43), and higher rates of substance abuse than the general population (44). Chronic levels of ill health exacerbate high levels of social disadvantage and poverty among this population (45) and psychosis has also been linked to a higher rate of utilization of specialist services (8, 9). In our study, we found that people with high levels of socioeconomic disadvantage were more likely to use emergency services for mental health reasons. This might be reflective of the lack of capacity to engage with health services or to manage comorbid illnesses; however, it could suggest differences in the availability of economic resources to access paid services such as GPs. They also accessed GPs less for mental health reasons, and had fewer family/social supports on which to draw. Consequently, the development of policy and practice that seeks to redress the socioeconomic and health inequalities created by this disadvantage should be an important focus for the improvement of mental health services.

This paper also highlights the complex relationship between socioeconomic disadvantage and poor health confronting individuals with psychosis. These results are also congruent with much of the social and health literature in the field that indicates that people with a psychotic illness are more likely to reside in public housing (46), receive a government income/pension, and experience economic disadvantage and social isolation (32, 47). While mental health services seek to provide strong clinical and non-clinical intervention for psychosis treatment, less focus is applied to enhancing illness recovery through socioeconomic engagement and participation. However, effective clinical mental health support and interventions for individuals require a coordinated and robust mental health system supported by social as well as health policy that places a priority on addressing socioeconomic disadvantage in mental health cohorts. Such a system would provide accessible treatment programs and linked pathways to illness recovery and diminish the pressure on the delivery of health services. Social disadvantage limits access to social services and limits participation in broader economic and cultural opportunities accessed by healthy populations. Residing in a disadvantaged community may also exacerbate socioeconomic disadvantage. Disadvantaged communities do not always have strong economic foundations, local organizational capacity, or community assets that can provide a social and health infrastructure required to sustain complex needs. Limited access to services, in turn, can further heighten vulnerability to poorer health, social and economic outcomes, and exacerbate marginalization within the communities in which psychosis populations live. Moreover, rates of psychosis are higher in disadvantaged communities (16), and illnesses such as schizophrenia are more prevalent in poor communities that also have higher levels of socioeconomic inequality (47).

### Limitations

There are some limitations with this study. As our data rely on retrospective self-report, the accuracy of the reporting of health service utilization, or health in general, may be affected by recall bias. Another limitation is that using an area-level measure, such as the SEIFA as a proxy measure of individual level disadvantage, assumes that the relationships observed for areas hold for individuals, and this may not be the case. Even in the most disadvantaged areas, there will be individuals who are less disadvantaged than others. However, given that the communities in this catchment area are resource poor, it is possible that area-level disadvantage could contribute substantially to individual disadvantage. It should also be noted that the cross-sectional nature of this study limits the conclusions that can be derived from the data, and while a cohort study would be more appropriate to assess long-term socioeconomic trajectories, it was beyond the scope and budget of the current study.

Additionally, further research into the links between psychosis, poor health, and health service utilization is required. How psychosis populations psychologically manage the intersections between these factors, including the need to maintain illness recovery, is a complex question that can only be addressed through comprehensive long-term research. Furthermore, 807 people did not respond to initial contact by SHIP researchers. The writers acknowledge that this might cause some degree of response bias in this study. This unrepresented cohort may have been either higher functioning or lower functioning or belong to certain ethnic groups who were unable to respond due to language difficulties. Others may have been deceased or be functionally illiterate.

## Conflict of Interest Statement

The authors report no conflicts of interest. The authors alone are responsible for the content and the writing of this article.

## Footnotes

^1^The northern suburbs of Adelaide consist of the cities of Playford, Salisbury, and Gawler, located in the northern region of metropolitan Adelaide, SA, Australia. The city of Gawler is located approximately 70 km from Adelaide’s CBD, and it is the most distant of the three council areas. The combined catchment area has an estimated resident population of approximately 230,000 within a radius of 815 km. The population density of the catchment is 432.4 residents/km.
